# Widely untargeted metabolomic profiling unearths metabolites and pathways involved in leaf senescence and N remobilization in spring-cultivated wheat under different N regimes

**DOI:** 10.3389/fpls.2023.1166933

**Published:** 2023-05-16

**Authors:** Zechariah Effah, Lingling Li, Junhong Xie, Benjamin Karikari, Aixia Xu, Linlin Wang, Changliang Du, Emmanuel Duku Boamah, Samuel Adingo, Min Zeng

**Affiliations:** ^1^ Department of Crop Science, State Key Laboratory of Arid Land Crop Science, Lanzhou, China; ^2^ College of Agronomy, Gansu Agricultural University, Lanzhou, China; ^3^ Department of Plant Genetic Diversity, Council for Scientific and Industrial Research (CSIR)-Plant Genetic Resources Research Institute, Bunso, Ghana; ^4^ Department of Agricultural Biotechnology, Faculty of Agriculture, Food and Consumer Sciences, University for Development Studies, Tamale, Ghana; ^5^ College of Forestry, Gansu Agricultural University, Lanzhou, China

**Keywords:** flavonoids, anthocyanins, grain yield, metabolite profile, nitrogen supply, postanthesis

## Abstract

Progression of leaf senescence consists of both degenerative and nutrient recycling processes in crops including wheat. However, the levels of metabolites in flag leaves in spring-cultivated wheat, as well as biosynthetic pathways involved under different nitrogen fertilization regimes, are largely unknown. Therefore, the present study employed a widely untargeted metabolomic profiling strategy to identify metabolites and biosynthetic pathways that could be used in a wheat improvement program aimed at manipulating the rate and onset of senescence by handling spring wheat (*Dingxi 38*) flag leaves sampled from no-, low-, and high-nitrogen (N) conditions (designated Groups 1, 2, and 3, respectively) across three sampling times: anthesis, grain filling, and end grain filling stages. Through ultrahigh-performance liquid chromatography–tandem mass spectrometry, a total of 826 metabolites comprising 107 flavonoids, 51 phenol lipids, 37 fatty acyls, 37 organooxygen compounds, 31 steroids and steroid derivatives, 18 phenols, and several unknown compounds were detected. Upon the application of the stringent screening criteria for differentially accumulated metabolites (DAMs), 28 and 23 metabolites were differentially accumulated in Group 1_vs_Group 2 and Group 1_vs_Group 3, respectively. From these, 1-O-Caffeoylglucose, Rhoifolin, Eurycomalactone;Ingenol, 4-Methoxyphenyl beta-D-glucopyranoside, and Baldrinal were detected as core conserved DAMs among the three groups with all accumulated higher in Group 1 than in the other two groups. Kyoto Encyclopedia of Genes and Genomes pathway analysis revealed that tropane, piperidine, and pyridine alkaloid biosynthesis; acarbose and validamycin biosynthesis; lysine degradation; and biosynthesis of alkaloids derived from ornithine, lysine, and nicotinic acid pathways were the most significantly (*p* < 0.05) enriched in Group 1_vs_Group 2, while flavone and flavonol as well as anthocyanins biosynthetic pathways were the most significantly (*p* < 0.05) enriched in Group 1_vs_Group 3. The results from this study provide a foundation for the manipulation of the onset and rate of leaf senescence and N remobilization in wheat.

## Introduction

The world is challenged to increase food production to meet the needs of increasing population growth, and this has become a major goal of researchers in the plant science community (Grote et al., 2021a). Achieving this objective is threatened by climate change, abiotic and biotic stresses, reduction in soil fertility, and decline in arable lands (Shiferaw et al., 2011; Oshunsanya et al., 2019; Savary et al., 2019). Wheat (*Triticum aestivum* L.) is currently the world’s most widely cultivated cereal and demand is expected to increase by 60% by 2050 (Vincent et al., 2022). It is the basic staple and food security crop accounting for over 60% of people’s daily calorific and protein needs (Grote et al., 2021b). To sustain food production, the use of synthetic fertilizers like nitrogen (N) has greatly enhanced crop production, but their long-term use and overuse to meet the present food demand can result in heavy deposit of nitrate in soils (Zhang et al., 2013), ammonia emission (Xu et al., 2019), and soil salinity (David et al., 2009) with detrimental effects on soil fauna and flora (Anas et al., 2020).

In order to understand the molecular mechanism underlying plant response to stresses as well as their development under optimal conditions, a number of omics strategies such as genomics, transcriptomics, proteomics, and phenomics have been applied to deepen our knowledge (Palmer et al., 2014; Francki et al., 2016; Zhen et al., 2016; Vergara-Diaz et al., 2020; Bai et al., 2023; Kaur et al., 2021; Singh et al., 2021; Effah et al., 2022). Over the past century, yield has been the key factor influencing crop innovation. The main drivers of high yield are improved agronomic practices (Whitcomb et al., 2014), the use of chemical fertilizers, and the development of molecular tools supporting plant breeding (Fernie and Schauer, 2009), and mineral nutrient availability is a crucial factor for achieving optimal yield (Kant et al., 2011; Gastal et al., 2015). Domestication of cereals has a lengthy history, and wheat in particular exhibits a decline in genetic diversity (Chen et al., 2012b). Elite wheat varieties are typically developed with high nitrogen fertilizer levels, which reduces diversity and variation in N use and remobilization efficiency across related populations (Shewry and Hey, 2015).

A balanced nutrient supply is one of the abiotic factors necessary for plant growth and development. Insufficient nutrient supply has numerous effects on plant physiology, including having a negative impact on crop performance and yield (Marschner, 1995). Nitrogen availability is essential for crop systems, particularly wheat, to produce their best yields (Hawkesford, 2014). In order to improve plant performance at a given nutrient supply through fertilizers or to breed or engineer plants to improve crop ability to deal with low nutrient availability, systems biology should be useful. Our understanding of the intricate molecular mechanisms behind the physiology of such abiotic nutrients is insufficient, and any additional system-wide information will help to eventually fill in the gaps in this regard.

The phenotype of a plant is influenced by interactions between its genotype and external environmental elements such as humidity, temperature, and light, but it is also influenced by the biochemical changes that occur within its cells as part of its homeostasis. Finding the precise alterations in metabolite concentrations involved in any biochemical pathway that characterizes the phenotype of a plant’s cells and tissues is exceedingly difficult in the case of plant metabolomics (Weckwerth, 2003). Metabolites are known to be the final products of cell activities that directly give a clue about the effect of environmental or physiological and pathogenic changes on plants (Shulaev et al., 2008). A total of 74 metabolites were reported during grain developmental stages in wheat with a correlation between metabolism of amino acids, carbohydrates, organic acids, amines, and lipids (Zhen et al., 2016). Metabolites such as organic acids (L-tartaric acid, a-hydroxyisobutyric acid, and 4-acetamidobutyric acid), sugars (melezitose, beta-D-lactose, D-sedheptulose 7-phosphate, 2-dexyribose 1-phosphate, and raffinose), and phenols (coniferin, curcumins, and feruloylputrescine) are reported to regulate post-harvest ripening of tomato fruit under low temperature (Bai et al., 2023), and these could be validated and used as biomarkers in practical crop improvement (Wen et al., 2014; Yan et al., 2021).

One of the physiological processes well known to affect grain yield/weight and quality is leaf senescence (Quirino et al., 2000; Watanabe et al., 2018; Paluch-Lubawa et al., 2021), which represents a phase of nutrient assimilation to remobilization to sink tissues (Masclaux et al., 2001; Li et al., 2017). Crops with delayed leaf senescence maintain leaf color and extend their photosynthetic competence, leading to a higher grain weight and yield (Spano et al., 2003; Borrell et al., 2014), while those with premature senescence of functional leaves at grain filling stages lead to reduced grain yield and quality (Spano et al., 2003). Applications of metabolomics in wheat study are highly valued and have great potential. To get more precise information from the wheat metabolome, however, a number of gaps and bottlenecks need to be resolved. With this in mind, the present study aimed to identify metabolites and biosynthetic pathways that could be used in a wheat improvement program targeted at manipulating the rate and onset of senescence in spring wheat by conducting metabolomic profiling of spring wheat (cultivar: *Dingxi 38*) flag leaves sampled from no-, low-, and high-N conditions in the semi-arid loess plateau of Gansu Province, People’s Republic of China. The flag leaves were sampled from three growth stages: anthesis, grain filling, and end of grain filling to give overall metabolic changes during these critical stages, which ultimately affect grain yield and quality (Djanaguiraman et al., 2020; Osman et al., 2021). The results of this study give an overview of metabolic changes involved in nutrient remobilization during the progression of leaf senescence in wheat, and lay a foundation in our attempt to manipulate leaf senescence and N remobilization *via* omics-assisted speed breeding for wheat improvement.

## Materials and methods

### Site description

This study was conducted in the Gansu Agricultural University’s Rainfed Experimental Station in Dingxi, Gansu Province, China (35_280N, 104_440E, elevation 1,971 m above sea level) from 2003 to date (March to July each year). In crop season, the average minimum and maximum air temperatures at the research location were −22 and 38°C, respectively, while the average precipitation was 390.7 mm year^−1^. With 2,480 h of sunshine for the crop season, the average crop season cumulative air temperature > 10°C was 2,240°C, and the average annual radiation was 5,930 MJ m^−2^. The site experienced a rise in the amount of rainfall from May and peaked in August and declined in September. Evaporation was three to four times higher than precipitation, with an average of 1,531 mm (coefficient of variation: 24.3%) for the crop season. The soil type at the site is a Huangmian sandy loam (Hocking and Stapper, 2001) and is classified as a Calcaric Cambisol (Hu et al., 2013). Flax (*Linum usitatissimum L*.) had been the previous crop, and the field has a long history of traditional farming with wheat pea rotation system. The chemical characteristics of the soil (at 0–30 cm depth) were found to be 3.88 kg ha^−1^ of total nitrogen (TN), 24.92 kg ha^−1^ of NH_4_-N, 12.72 kg ha^−1^ of NO_3_-N, pH 8.33, 8.3 kg ha^−1^ of total phosphorus (P), 4.53 kg ha^−1^ of accessible P, and 82.68 kg ha^−1^ of total potassium (K).

### Samples and sampling

Spring-cultivated wheat (*Dingxi 38*) was planted under five N fertilizer (urea) treatments, i.e., 0, 52.5, 105, 157.5, and 210 kg N ha^−1^ represented by N1, N2, N3, N4, and N5, respectively. Flag leaves were sampled from N1, N2, and N5 to represent no-, low-, and high-N conditions at anthesis and 14 and 28 days after anthesis (DAA). The three representative samples from no-N plots at the three stages were named WNR1, WNR7, and WNR13 for anthesis, 14 DAA, and 28 DAA. In the same order, samples from low-N plots were denoted WNR2, WNR8, and WNR11, while those from high-N plots were named WNR5, WNR9, and WNR14. At each sampling stage, the well-labeled tubes with each group sample were quickly placed in liquid nitrogen, stored on ice, and transported to the laboratory. Then, the samples were stored in a −80°C ultralow-temperature refrigerator until all the three sampling stages were done.

### Measurement of leaf chlorophyll content

Using a soil-plant analysis development chlorophyll meter (SPAD-502, Konica Minolta Inc., Osaka, Japan), the amount of chlorophyll per unit area of flag leaves was calculated from anthesis to the soft dough stage at 3, 7, 10, 14, 18, 21, and 28 DAA from 22 June to 22 July 2020. The meter readings were taken on the second completely expanded leaf, halfway along the leaf blade, and halfway between the central vein and the leaf edge; the concentration of chlorophyll (Chl) content per unit area was calculated in attached leaves. On leaves that were later plucked for the metabolite analysis, measurements were also taken. An approved substitute for leaf nitrogen concentrations is leaf greenness (Lawlor, 2002; Foyer et al., 2003).

### Sample and metabolite extraction process

Methanol/chloroform extraction, as previously described, was used to extract the metabolites (Erban et al., 2007). The leaves were first freeze-dried and then ground to powder using a grinder (MM 400; Retsch, Germany) with a zirconia bead for 1.5 min at 30 Hz. From 0.1 g of freeze-dried plant material, metabolites were extracted using 1 ml of 70% aqueous methanol at 4°C overnight, and vortexed three times during the period to enhance the extraction efficiency, 30 ml of nonadecanoic acid methylester (2 mg/ml stock in chloroform), and 30 ml of a pre-mixture of sorbitol (0.2 mg/ml methanol) and D-(−)-isoascorbic acid (0.5 mg/ml in H_2_O) for quantitative internal standardization. Chloroform (200 µl) was added to the polar phase after the extract had been heated for 15 min at 70°C. After shaking the mixture at 37°C for an additional 5 min, 400 µl of water was added to it. The mixture was then vigorously vortexed before being centrifuged to separate the polar and non-polar phases. A speed-vac was used to dry 100-µl aliquots at a time. We assessed the amounts of primary metabolites and ions in dried extracts. Then, the extract was centrifuged at 10,000 *g* for 10 min, and finally, the supernatant was filtered through a microporous membrane (0.22 μm pore size; ANPEL, Shanghai, China) before ultrahigh-performance liquid chromatography–tandem mass spectrometry (UHPLC-MS/MS) analysis.

### Identification and analyses of metabolites

Widely untargeted metabolomic profiling of samples was conducted by Gene pioneer Biotechnology Co., Ltd. (Su ICP No. 15038772-1; 9 Weidi Road, Xianlin University Town, Qixia District, Nanjing) following the protocol earlier published by Want et al. (2010) with minor modification. Briefly, the chromatographic separation was undertaken *via* an Accucore Hydrophilic interaction liquid chromatography column with dimensions 50 × 2.1 mm and 2.6 μm (Thermo Fisher ScientificTM, United States) fitted to the Thermo Scientific UHPLC system. Metabolites were eluted from the column through a gradient mobile phase that comprised phase A (0.1% formic acid, 10 mM ammonium acetate, and 95% acetonitrile) and phase B (made up of 0.1% formic acid, 10 mM ammonium acetate, and 50% acetonitrile), at a flow rate of 0.3 ml min^−1^. A volume of 5 μl per sample was added after equilibration. The temperature of the column was maintained at 40°C, while the auto-sampler was kept at 4°C. The linear gradient elution procedure followed 2% B for 0–1 min, from 2% B at 1 min to 50% B at 17 min until 17.5 min, thereafter returning to the initial gradient conditions (2% B) at 18 min, followed by maintaining at 2% B for a period of 18–20 min. In order to prevent the influence caused by the detected signal fluctuation of the apparatus, samples were randomly injected. A quality control (QC) sample followed by a blank sample was injected after every three experimental sample injections.

The electrospray ionization–mass spectrometry (ESI-MS) experiments were carried out on a Thermo Q ExactiveTM HF-X mass spectrometer with a spray voltage of 3.2 kV in both positive and negative modes. The sheath gas flow rate was set at 35 arbitrary units, while that of auxiliary was set at 10 arbitrary units. In addition, the capillary temperature was set at 320°C, and the MS analysis alternated between MS full scans and data-dependent MS/MS scans with dynamic exclusion. Finally, the mass scan range was selected from 100 to 1500 m/z at a scan rate of 40 Hz.

### Data collection and processing

The total ion chromatograms of the nine samples were extracted after detection by UHPLC-MS/MS. The acquired raw MS files were processed via the Compound Discoverer (Thermo Fisher Scientific) software for data pretreatments covering peak identification, alignment, feature extraction, and area normalization, running separately under positive and negative ionization mode following the procedure outlined by Yu et al. (2018).

To begin with, screening for retention time (RT), mass-to-charge ratio (m/z), and other parameters as well as the peak alignment of different samples was carried out according to the RT deviation of 0.2 min and the mass deviation of 5 ppm in order to make the identification more accurate. Subsequently, the peak extraction was conducted according to the following criteria: mass deviation = 5 ppm, signal strength deviation = 30%, signal-to-noise ratio = 3, and minimum signal strength = 100,000. Moreover, the peak area was then quantified. The mass spectrometry matrix data containing sample names, m/z–RT pairs, and ion intensity information were generated and exported. The target ions were then integrated to predict the molecular formula and compared against the Japan Chemical Substance Dictionary (NIKKAJI) (Kimura and Kushida, 2015), Chemical Entities of Biological Interest (ChEBI) (Degtyarenko et al., 2007), PubChem (Kim et al., 2016), Kyoto Encyclopedia of Genes and Genomes (KEGG) both compound name and pathway (Kanehisa et al., 2016), and class assignment and ontology prediction using mass spectrometry (CANOPUS) (Dührkop et al., 2021) online databases for identification and confirmation of the compounds. The background ions were eliminated with blank samples, and the quantitative results were then normalized with QC samples. The identification and quantitative results from the data were obtained and used for further statistical analysis.

### Statistical analyses

Multivariate statistical analyses: principal component analysis (PCA) and orthogonal projections to latent structures-discriminant analysis (OPLS-DA) of ion intensities of metabolites detected in the no, low, and high N renamed as Groups 1, 2, and 3, respectively, were performed with SIMCA-P software (v13.0, Umetrics, Umea, Sweden) after Pareto scaling in order to show the differences in the metabolic composition among the nine samples from the three groups. The reliability of the OPLS-DA model was verified by permutation test, which was used to assess whether the model was overfitted.

To determine the differentially accumulated metabolites (DAMs) between the Group 1 and either Group 2 or 3 samples, variable importance in the projection (VIP) > 1, *p*-value < 0.05, and fold change (FC) > 1.2 or FC < 0.83 were adopted for screening (Saccenti et al., 2014). The volcano and heatmap hierarchical clustering plots were generated with the *DESeq2* (Love et al., 2014) and *pheatmap* (Kolde, 2012) packages in R to visualize the metabolite profiles and reveal the relationship between the metabolites and samples. To determine the extent of relationship between metabolites in the pairwise group comparison, the *corrplot* package in R was used to generate and visualize Pearson correlation coefficients between metabolites (Wei et al., 2017).

Aside from the above, biosynthetic pathway enrichment was conducted according to KEGG *via* MetaboAnalyst based on the ion intensity of Group 2 or 3 relative to Group 1 with the threshold of *p* < 0.05 and finally bubble plots for two groups with the top 20 pathways were generated by *ggplot2* package in R (Wickham et al., 2016). A Venn diagram and a histogram of extent regulation of DAMs between the two pairwise groups were generated by TBtool (Chen et al., 2020) and GraphPadPrism (GraphPad Software, Inc., www.graphpad.com), respectively.

## Results

### Effect of N fertilization on chlorophyll content in leaves

**Figure 1:**
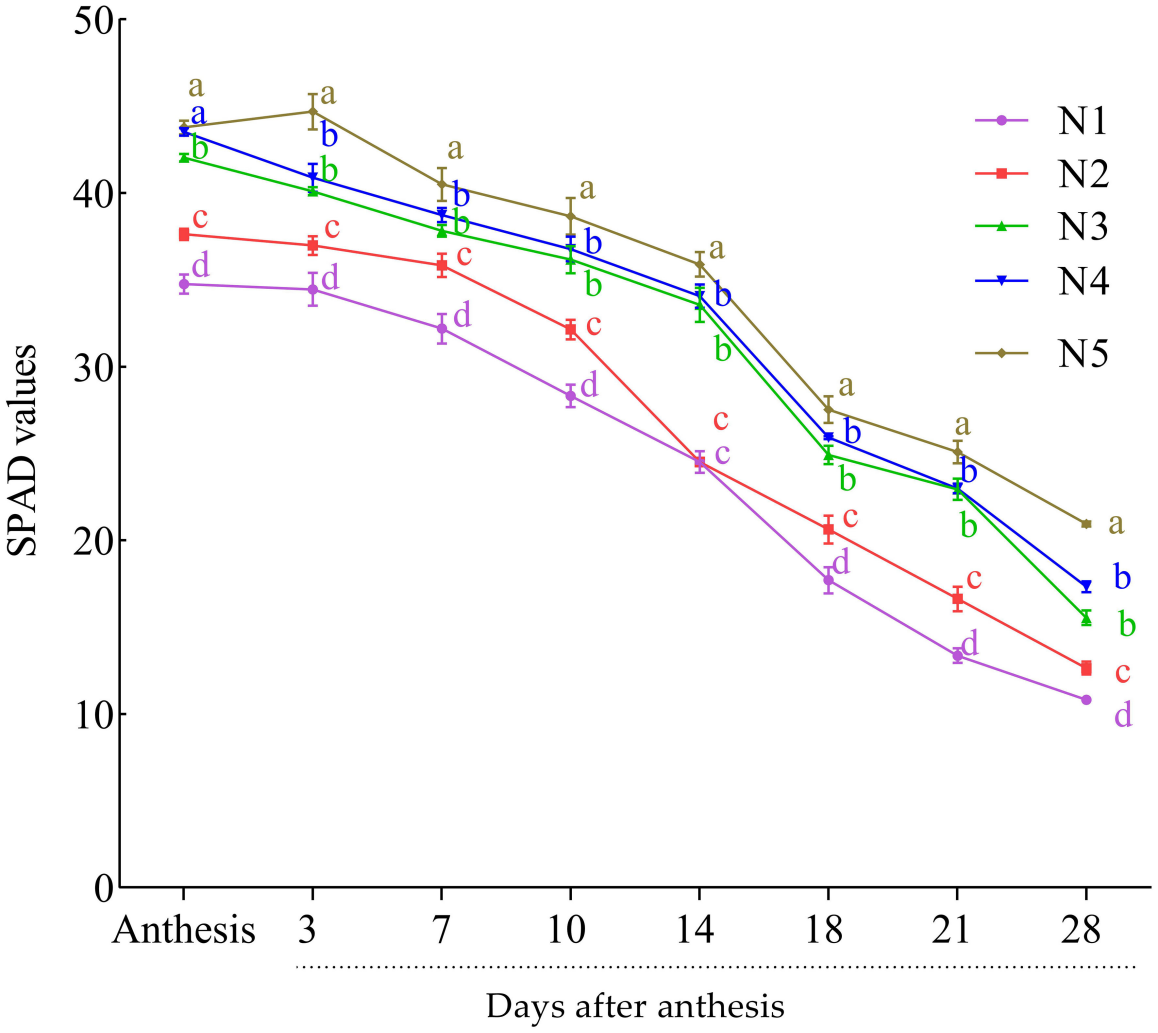
Effect of nitrogen rates on SPAD value at post-anthesis growth stages of the wheat cultivar (average of the 2 years: 2019 and 2020). Results are mean ± standard error (*n* = 3). Treatment comprised N at different rates: 0 kg ha^−1^ (N1), 52.5 kg ha^−1^ (N2), 105 kg ha^−1^ (N3), 157.5 kg ha^−1^ (N4), and 210 kg ha^−1^ (N5). Alphabets at each sampling time (days) indicate the level of difference among the five levels N used in this study. N level with the same or a common alphabet indicate no significant difference (P-vale >0.05), while those with different alphabet indicate significant difference (P-value<0.05).

The SPAD values of leaves were taken as an indicator of chlorophyll content from anthesis to the end of grain filling (28 DAA). The highest SPAD values were recorded at the anthesis stage and slowly declined towards maturity (**Figure 1**). At anthesis and post-anthesis, the SPAD value was the highest at the higher N rate (N4 and N5) and then declined with the decrease in N rate and was the lowest at control. The relative decline of the chlorophyll from anthesis to maturity was 52% under N5 treatment, while in the control and N2, the decline was 69% and 66.4%, respectively. However, no significant difference was observed between N4 and N3 in all the growth stages except at the end of grain filling.

### Metabolites detected among the flag leaves from three N conditions at three different stages

In our earlier study, it was observed that N rate alters leaf senescence and N remobilization in spring-cultivated wheat, *Dingxi 38* (Effah et al., 2022); therefore, a UHPLC-MS/MS-based untargeted metabolomic approach was performed to profile the metabolites in flag leaves of spring-cultivated wheat, *Dingxi 38*, sampled from three N conditions (no, low, and high N) and three stages (anthesis, 14 DAA, and 28 DAA). The nine samples (3 N conditions × 3 stages) were grouped into Groups 1 (WNR1, WNR7, and WNR13), 2 (WNR2, WNR8, and WNR11), and 3 (WNR5, WNR9, and WNR14) under no, low, and high N, respectively. From these, we detected a total of 826 metabolites (**Supplementary Table S1**), of which 576, 495, 489, 365, 355, 335, and 291 were successfully confirmed in KEGG compound name (Kanehisa et al., 2016), the Human Metabolome Database (HMDB) (Wishart et al., 2018), CANOPUS (Dührkop et al., 2021), PubChem (Kim et al., 2016), NIKKAJI (Kimura and Kushida, 2015), ChEBI (Degtyarenko et al., 2007), and KEGG pathway (**Supplementary Figure S1**). The successful confirmation of detected compounds on these public databases gives credence to our results.

Among the classes of compounds, 107 are flavonoid and its associated compounds (2-arylbenzofuran flavonoids, flavonoid, and isoflavonoid), 51 are prenol lipids, 37 are fatty acyls, 37 are organooxygen compounds, 31 are steroids and steroid derivatives, 18 are phenols, and others such as carbohydrates and carbohydrate conjugates, phenylpropanoic acids, phenylpropanoids, and polyketides (**Supplementary Figure S1**). The dominance of some classes of compounds gives clues to their potential role in regulating spring wheat under different N regimes.

**Figure 2:**
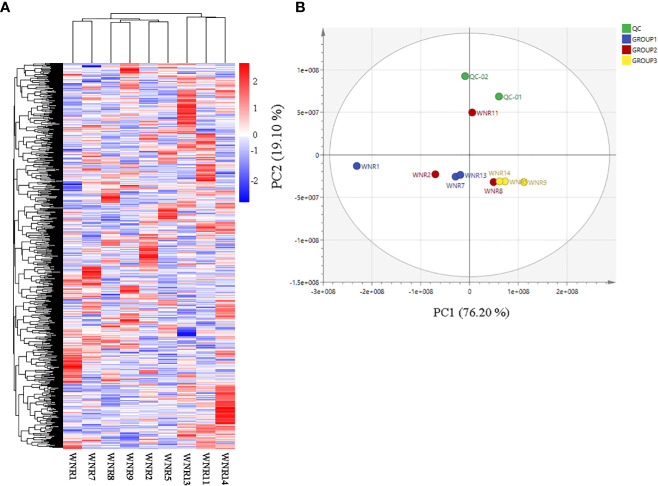
Quality assessment of metabolites based on their ion intensities from three nitrogen conditions. **(A)** Hierarchical heatmap clustering of all metabolites. **(B)** Principal component analysis. Three N conditions: no, low, and high nitrogen designated as Groups 1 (WNR1, WNR7, and WNR13), 2 (WNR2, WNR8, and WNR11), and 3 (WNR5, WNR9, and WNR14), respectively, and three stages [anthesis—WNR1, WNR2, and WNR5; grain filling period (14 days after anthesis)—WNR7, WNR8, and WNR9; and end of grain filling period—WNR11, WNR13 and WNR14]. QC represents quality control sample comprising the mixture of the three groups.

Based on the ion intensities of the 826 metabolites, we performed hierarchical heatmap clustering with the *pheatmap* package in R. The nine samples clustered into 2 clusters with Cluster 1 comprising samples from anthesis (WNR1, WNR2, and WNR5) and the grain filling stage (WNR7, WN8, and WNR9), while Cluster 2 consisted of only samples from the end of the grain filling stage (**Figure 2A**), suggesting that similar compounds are produced by wheat plants at either of the three stages (anthesis, grain filling, and end of grain filling) or three conditions (no, low, and high N rate), but differed in their intensity. In addition, PCA revealed that the first and second PC axes account for 95.30% variability among the nine samples (**Figure 2B**).

Taken together, the detected compounds, their confirmation on prominent public databases, and variability by cluster and PC analyses pinpoint the use of metabolites in regulating spring-cultivated wheat under different N rates. This called for the current study to discover and dissect the metabolites and pathways involved in regulating spring-cultivated wheat under different N rates.

### Identification of differentially accumulated metabolites

The 826 metabolites detected in the nine flag leaf samples were subjected to differential accumulation analysis with Group 1 (no-N condition [WNR1, WNR7, and WNR13]) relative to either Group 2 (low-N condition [WNR2, WNR8, and WNR11]) or Group 3 (high-N condition [WNR5, WNR9, and WNR14]) samples *via* OPLS-DA, VIP > 1, *p*-value < 0.05, and FC > 1.2 or FC < 0.83 (**Figures 2A, B**). The goodness of prediction models of Group 1_vs_Group 2 and Group 1_vs_Group 3 samples caused 100% and 97.20% variability in the pairwise comparisons, respectively (**Supplementary Figures S2A, B**).

**Figure 3:**
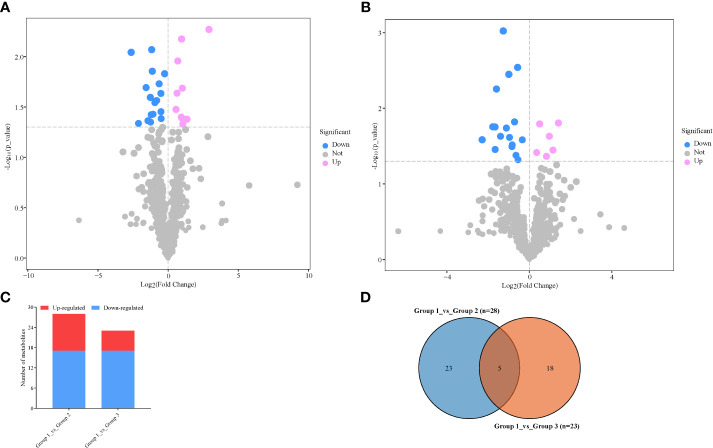
Differentially accumulated metabolites (DAMs) detected in samples from no nitrogen (Group 1) relative to either low nitrogen (Group 2) or high nitrogen (Group 3). **(A)** Volcano plot in Group 1_vs_Group 2. **(B)** Volcano plot in Group 1_vs_Group 3. Each point in the volcano plot represents a metabolite, the abscissa represents the fold change of each substance in the group compared to each other (take the logarithm with the base 2), and the ordinate represents the *p*-value of the Student’s *t*-test (take 10 as the logarithm of the base). The size of the scatter represents the importance in projection (VIP) value of the orthogonal partial least squares discriminant analysis model; the larger the scatter, the greater the variable VIP value. Scattered colors represent the final screening results; significantly upregulated metabolites are shown in red, significantly downregulated metabolites are shown in blue, and non-significantly different metabolites are shown in gray. **(C)** Extent of regulation of DAMs. **(D)** Venn diagram of DAMs between the two pairwise comparisons.

From the above stringent criteria, a total of 28 and 23 DAMs were detected in Group 1_vs_Group 2 (VIP = 1.50–1.71) and Group 1_vs_Group 3 (VIP = 1.49–1.76), respectively (**Figures 3A–D**; **Supplementary Table S2A**). Among those DAMs in Group 1_vs_Group 2, 17 and 11 accumulated higher and lower respectively (**Figure 3C**; **Supplementary Table S2A**). The up-accumulation of higher numbers of DAMs observed in Group 1 could be because of stressful conditions by the no-N treatment, which is responsible for influencing the number of metabolites for survival. The top three metabolites with the lowest FC between Group 1 and Group 2 include diethanolamine, 3-aminoisobutanoic acid, and 1-O-caffeoylglucose, while bergaptol, avenanthramide A, baptifoline, and (2E)-decenoyl-ACP;L-pipecolic acid;pipecolic acid accumulated at least 2.07-fold higher in Group 2 than in Group 1 (**Supplementary Table S2A**).

On the other hand, 17 metabolites accumulated higher under no-N conditions than under high-N conditions, while 6 metabolites had higher accumulation under high-N conditions than under low-N conditions (**Figure 3C**; **Supplementary Table S2B**). This highlights that N stress stimulates the accumulation of a number of metabolites essential to survival, hastens delay senescence, and reduces N remobilization. The top seven differentially accumulated metabolites with the least −log_2_FC values are as follows: 1-O-caffeoylglucose > anisatin > 6,7-dehydroferruginol > baldrinal > tricin > tetrahydrocurcumin > astragalin (**Supplementary Table S2B**). The six metabolites with the highest −log_2_FC are as follows: 1-(4-Hydroxyphenyl) propan-1-one < phendimetrazine < proline betaine < azithromycin < lupeol< sclareol (**Supplementary Table S2B**).

To identify conserved DAMs (CDAMs), five CDAMs (1-O-caffeoylglucose, rhoifolin, eurycomalactone;ingenol, 4-methoxyphenyl beta-D-glucopyranoside, and baldrinal) among the three conditions (no, low, and high N), all accumulated higher in the no-N condition than in either the low- or high-N condition (**Table 1**), suggesting that spring wheat increases the abundance of some of the metabolites to survive under the no-N condition (Law et al., 2018).

**Table 1:** Average ion intensities of the five common differentially accumulated metabolites detected among the three groups.

Metabolite	Class ^a^	Group 1^b^	Group 2^c^	Group 3^d^
1-O-Caffeoylglucose	Carbohydrate	1.24E-04	4.19E-05	2.55E-05
Rhoifolin	Flavonoid	4.58E-03	3.20E-03	3.61E-03
Eurycomalactone;Ingenol	Diterpenoids	1.14E-04	6.45E-05	7.76E-05
4-Methoxyphenyl beta-D-glucopyranoside	Phenol	6.23E-05	2.59E-05	3.51E-05
Baldrinal	Miscellaneous	9.16E-05	3.86E-05	2.90E-05

^a^ Assignment and ontology prediction using mass spectrometry (CANOPUS) (Dührkop et al., 2021). ^b^ Comprised WNR1, WNR2, and WNR5 samplings taken at anthesis. ^c^ Comprised WNR7, WNR8, and WNR9 samplings taken at the grain filling period [14 days after anthesis (DAA)]. ^d^ Comprised WNR11, WNR13, and WNR14 samplings taken at the end of the grain filling period (28 DAA).

### Functional analyses of differentially accumulated metabolites

The DAMs detected were annotated by the KEGG database, and the main enriched metabolic pathways of DAMs were analyzed. The results revealed that one compound—(2E)-decenoyl-ACP;L-pipecolic acid; pipecolic acid (carboxylic acids and derivatives; amino acid and derivative)—involved in amino acid metabolism was enriched on lysine degradation (map00310), tropane, piperidine, and pyridine alkaloid biosynthesis (map00960), and biosynthesis of alkaloids derived from ornithine, lysine, and nicotinic acid (map01064); these together with acarbose (carbohydrates and carbohydrate conjugates) involved in acarbose and validamycin biosynthesis (map00525) were significantly enriched in Group 1_vs_Group 2 (**Supplementary Table S3A** and **Figure 5A**). These two compounds, (2E)-decenoyl-ACP;L-pipecolic acid;pipecolic acid and acarbose, accumulated higher in Group 2 than in Group 1, suggesting their possible involvement in the delayed senescence and higher N remobilization observed in Group 2 (**Figures 6A, B**).

Plants are sessile organisms and, as a result, develop different methods for protection against stressful conditions of the surroundings, including abiotic stress like limited/no-N condition. One prominent mechanism is the production of secondary metabolites such as flavonols, anthocyanins, and catechins (Gutierrez et al., 2017; Zandalinas et al., 2017). Interestingly, two anthocyanin biosynthetic-based metabolites (cyanidin 3-O-rutinoside and peonidin 3-O-glucoside) (**Figures 6C, D**) and two flavone and flavonol biosynthetic-based metabolites (map00944) (rhoifolin and astragalin, alternatively known as apigenin 7-O-neohesperidoside and Kaempferol 3-O-glucoside) (**Figures 6E, F**) accumulated higher in Group 1 than in Group 3, pointing out that the wheat plants under the no-N condition activate derivative pathways of phenylpropanoid biosynthesis. The number of metabolic pathways discovered could be targeted for metabolic engineering to delay leaf senescence while optimizing N remobilization in spring-cultivated wheat.

We further performed Pearson correlation analyses among the DAMs detected in each pairwise comparison, and the results of correlation coefficients (*r*) are shown in **Figures 7A, B** and **Supplementary Tables S4A, B**. It was observed that Acarbose negatively correlated with diethanolamine (*r* = −0.902, *p* = 0.014), 4-methoxyphenyl beta-D-glucopyranoside (*r* = −0.860, *p* = 0.028), and rhoifolin (*r* = −0.9866, *p* = 0.026) (**Figure 7A**; **Supplementary Table S4A**). The latter also negatively correlated with sinalbin (*r* = −0.896, *p* = 0.016). These strong negative correlated compounds could be targeted to manipulate acarbose and rhoifolin to increase yield under the no-N condition and regulate leaf senescence. rhoifolin, on the other hand, had a strong significant positive correlation with fucoxanthin (*r* = 0.942, *p* = 0.005), terpinolene (*r* = 0.961, *p* = 0.002), and picrocrocin (*r* = 0.963, *p* = 0.002), while acarbose and 1-methy-L-histidine had *r* = 0.963 at *p* = 0.002.

Under no N (Group 1) relative to high N (Group 3), astragalin/kaempferol 3-O-glucoside involved in flavone and flavonol biosynthesis increased significantly with 6,7-dehydroferruginol, herniarin, metanephrine, peonidin-3-glucoside, and tricin with *r* = 0.912–0.930 with *p* < 0.05, while astragalin/kaempferol 3-O-glucoside decreased in abundance as lupeol and phendimetrazine decreased significantly (**Figure 7B**; **Supplementary Table S4B**). In addition, cyanidin 3-rutinoside and biorobin implicated in anthocyanin biosynthesis significantly increased with the increase in 1-O-caffeoylglucose, 4-methoxyphenyl beta-D-glucopyranoside, and metanephrine (**Figure 7B**; **Supplementary Table S4B**). These give clues about the association between metabolites, which could be validated, and a few selected for biomarkers to aid omics-based wheat senescence and N remobilization improvement programs.

### Effect of N fertilization on chlorophyll content in leaf

The primary objective of breeding is to increase yield, which is also necessary given the impending population growth, but this is only accomplished through small, gradual steps. The genetic makeup of the crop and the environmental factors, such as the availability of nutrient ions, both affect the yield in a multifactorial manner. A greater molecular understanding of the physiological and biochemical processes in agricultural plants holds the potential to bring a knowledge-based component to plant breeding in addition to the extremely efficient breeding and selection procedures. In order to find features or processes that are important under natural multifactorial situations, it is also vital to research such processes under field conditions. The goal of the experiment was to identify the metabolites of field-grown spring wheat under various N conditions targeted at manipulating the rate and onset of senescence by conducting metabolomic profiling of spring wheat leaf.

The results of the current study indicated that the highest N rate had the highest SPAD (chlorophyll) (**Figure 1**). In order to evaluate N level and N-use efficiency and to boost grain yield, chlorophyll content has been widely used in most cereal plants, including wheat (Singh and Ali, 2020; Ghosh et al., 2020). The chlorophyll content decreased from anthesis to maturity; in particular, more decrease was observed at the late grain filling stages (**Figure 1**). The first declines in chlorophyll content signaled the beginning of senescence and the transport of solutes to the growing grain simultaneously with the drop in nitrate content in the leaves at 7 DAA. The beginning of senescence usually leads to massive remobilization of phloem-mobile nutrients from the senescing plant parts to a developing sink, such as seeds and grains of monocarpic crops. Senescence is accelerated by low N levels, and this is accompanied by earlier (plastidial) protein breakdown because of sink demand (Yoshida, 2003). According to Martre et al. (2006), high N levels can delay senescence because more stored inorganic or organic nitrogen can be used to meet sink demand, resulting in faster rates of continuous photosynthesis. The senescing organs of the parent plant provide a major portion of the transportable micro- and macronutrients that wheat grains need to grow, with root absorption providing a smaller portion (Kichey et al., 2007). Senescence and nutrient remobilization processes that occur during the reproductive stage are therefore more crucial for understanding the mechanisms that regulate plant productivity than organ senescence (sequence leaf senescence) activities that take place during vegetative plant growth and development (Martre et al., 2006).

The discovery of the genes responsible for no- or low-N tolerance in spring wheat will contribute to the understanding of the molecular mechanisms behind no- or low-N tolerance, as well as the genetic improvement of spring wheat through marker-assisted selection or gene transformation (Feller et al., 2008).

### Differentially accumulated metabolites and their functional analysis relative to leaf senescence and N remobilization

Throughout breeding history, wheat genetic diversity endured a significant contraction (Zamir, 2001; Fu, 2015). Furthermore, plants tend to maintain nitrogen homeostasis for as long as possible through a variety of adaptation mechanisms, such as increased uptake of N, altered mobilization patterns, decreased growth, and the ability to maintain metabolite concentrations under abiotic stress conditions, as demonstrated for Arabidopsis under N-depletion (Heyneke et al., 2017). This makes studying leaf senescence after anthesis a prerequisite to understanding the physiological and genetic basis of photosynthesis, nutrient remobilization, and the rules of crop yield and quality of great value to the practice of crop improvement (Yuan et al., 2022). With the above in mind, the present study applied widely untargeted metabolomic profiling to uncover metabolites that may be involved in leaf senescence and N remobilization with three levels of N fertilization regimes (no, low, and high N). The use of untargeted metabolomics is well known to have a comparative advantage over targeted metabolomics. Briefly, untargeted metabolomics is a strategy that focuses on global detection and relative quantitation of small-molecule metabolites to dissect both known and unknown metabolic changes that accompany changes in the environment (Gomez-Gomez et al., 2019; Mehta et al., 2020). Using this strategy allows for simultaneous detection and quantification of a wide range of compounds that cover known and unknown classes of compounds, which make it ideal for the detection of unexpected changes or unknown information in metabolite levels (Gomez-Gomez et al., 2019; Li et al., 2019). By widely untargeted metabolomic profiling, we detected a total of 826 metabolites, of which larger portions were successfully mapped onto seven credible public databases (**Supplementary Table S1**; **Supplementary Figure S1**). The most prominent known class of compound detected was flavonoid and its associated compounds such as arylbenzofuran flavonoids, flavonoid, and isoflavonoid. It is not surprising as flavonoid accumulation is reported to increase in plants growing in soils with low N concentration (Bongue Bartelsman and Phillips, 1995; Aires et al., 2006; Ibrahim et al., 2012). One prominent mechanism adopted by stressful plants is the production of secondary metabolites such as flavonols, anthocyanins, and catechins (Dixon and Paiva, 1995; Zandalinas et al., 2017).

**Figure 4:**
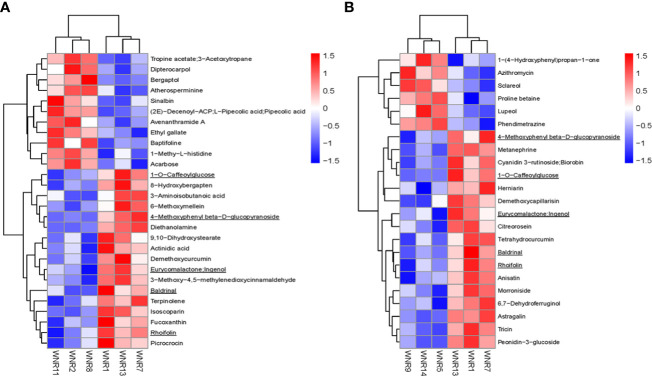
Heatmap clustering of differentially accumulated metabolites among the three groups based on their ion intensities. **(A)** Group 1_vs_Group 2. **(B)** Group 1_vs_Group 3. Five common DAMs are underlined. Three groups comprised no, low, and high nitrogen designated as Groups 1, 2, and 3, respectively. WNR1, WNR2, and WNR5 samplings were taken at anthesis; WNR7, WNR8, and WNR9 samplings were taken at the grain filling period [14 days after anthesis (DAA)]; and WNR11, WNR13, and WNR14 sampling were taken at the end of the grain filling period (28 DAA).

Upon application of the strict screening threshold of OPLS-DA, VIP > 1, *p* < 0.05, and FC > 1.2 or FC < 0.83, it was observed that the no-N condition (Group 1) induced and accumulated a higher number of metabolites as a survival mechanism. It has been reported that secondary metabolites such as phenylpropanoids and their derivatives, i.e., flavonoids, anthocyanins, coumarins, lignin building blocks, and tannins are an essential group of compounds necessary for plant acclimation and survival to varying environmental conditions including limited N conditions (Fraser and Chapple, 2011). For example, astragalin/kaempferol 3-O-glucoside, a flavonoid compound, accumulated nearly 60% higher in the no-N condition (Group 1) than in the high-N condition (Group 3) (**Supplementary Table S2B**). In addition, 1-O-caffeoylglucose, a well-known high-energy glucose ester potentially utilized as a donor molecule during the formation of diverse hydroxycinnamic acid O-esters in plants (Mock and Strack, 1993; Lim and Bowles, 2004; Le Roy et al., 2016), accumulated 79.50% and 387.91% higher in the no-N condition plants (Group 1) than in the low-N (Group 2) and high-N (Group 3) condition plants, respectively (**Figure 4**; **Table 1**). This suggests that stressed wheat plants under no N tend to spend a lot of energy for survival and maintenance of their physiological role. These compounds may be responsible for lower photosynthetic capacity observed under limited N conditions compared with Group 3. This corroborates with the reports of Chen et al. (2012a) and Hao et al. (2021) that higher accumulation of flavonoid compounds may affect the photosynthetic capacity of leaves.

**Figure 5:**
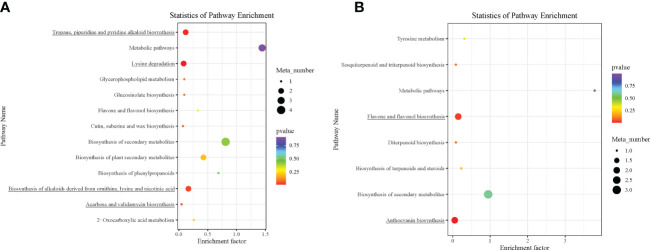
Kyoto Encyclopedia of Genes and Genomes (KEGG) pathway enrichment analysis bubble plot of differentially accumulated metabolites. **(A)** Group 1_vs_Group 2. **(B)** Group 1_vs_Group 3. Each row in the figure represents a KEGG pathway. The abscissa is the enrichment factor. The larger the enrichment factor, the more significant the enrichment level of differential metabolites in this pathway. The color of the dots represents the *p*-value, and the size of the bubbles represents the number of differential metabolites annotated in that pathway. Those underlined had *p* < 0.05.

**Figure 6:**
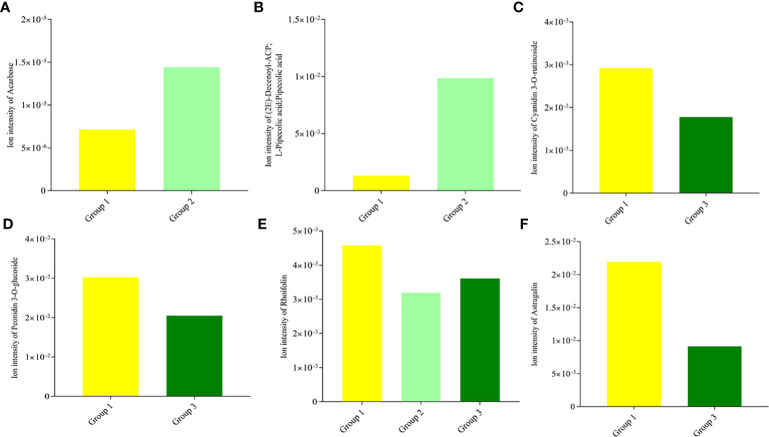
Ion intensities of differentially accumulated metabolites enriched in significantly *(p* < 0.05) associated Kyoto Encyclopedia of Gene and Genome pathway analyses. **(A)** Acarbose compound in acarbose and validamycin biosynthesis (map00525). **(B)** (2E)-decenoyl-ACP;L-pipecolic acid;pipecolic acid in amino acid metabolism [lysine degradation (map00310), tropane, piperidine, and pyridine alkaloid biosynthesis (map00960), and biosynthesis of alkaloids derived from ornithine, lysine, and nicotinic acid (map01064)]. **(C)** Cyanidin 3-O-rutinoside in anthocyanins biosynthesis (map00942). **(D)** peonidin 3-O-glucoside in anthocyanins biosynthesis (map00942). **(E)** Rhoifolin/apigenin 7-O-neohesperidoside in flavone and flavonol biosynthesis (map00944). **(F)** Astragalin/kaempferol 3-O-glucoside in flavone and flavonol biosynthesis (map00944).

In addition to the above, comparison of Group 1 to Group 2 presented compelling evidence for the possible basis for the higher grain yield and quality observed in Group 2 than in Group 1. One carbohydrate and its conjugate—acarbose—was implicated in acarbose and validamycin biosynthesis (map00525), and one carboxylic acid and derivative/amino acid and derivative—(2E)-decenoyl-ACP;L-pipecolic acid;pipecolic acid—were involved in amino acid metabolism-related biosynthetic pathways: lysine degradation (map00310); tropane, piperidine, and pyridine alkaloid biosynthesis (map00960); and biosynthesis of alkaloids derived from ornithine, lysine, and nicotinic acid (map01064) (**Figures 6A, B**) (Yang et al., 2021). Consistent with previous studies, amino acid metabolism is well documented to be involved in leaf senescence and N remobilization (Li et al., 2017). Acarbose and (2E)-decenoyl-ACP;L-pipecolic acid;pipecolic acid could be targeted to increase grain yield and quality as biomarkers for omics-based crop improvement program in wheat (Steinfath et al., 2010).

**Figure 7:**
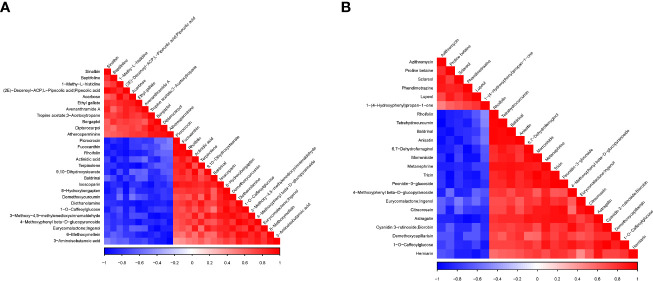
Association analysis heatmap of differentially accumulated metabolites detected in the pairwise comparisons. **(A)** Group 1_vs_Group 2. **(B)** Group 1_vs_Group 3. For the different metabolites represented vertically and diagonally in the figure, the color represents the level of correlation between different substances. The darker the color, the higher the correlation. at P-value < 0.05, while those with lighter color indicate no significant correlation (P-value>0.05).

Plant pigment is one of the determinants of photosynthetic capacity and efficiency. Some flavonoids play an important role in plant development and defense. Flavonoids constitute one of the main pigments in plants, such as anthocyanins (red, orange, blue, and purple pigments); chalcones and aurones (yellow pigments); and flavonols and flavones (white and pale-yellow pigments), which impart on plants a wide variety of colors (Grotewold, 2006). This was evidenced in our current study as two anthocyanin biosynthetic-based metabolites (cyanidin 3-O-rutinoside and peonidin 3-O-glucoside) (**Figures 6C, D**) and two flavone and flavonol biosynthetic-based metabolites (rhoifolin/apigenin 7-O-neohesperidoside and astragalin/kaempferol 3-O-glucoside) (**Figures 6E, F**) accumulated higher in Group 1 than in Group 3, highlighting the role of flavonoid compounds in modulating leaf senescence under N-deficient conditions. The excess accumulation of flavonoid compounds under limited N conditions according to Moreland and Novitzky (1998) likely involves the interruption of the electron transport in the photosystem II reaction center through the disruption of the function of the secondary electron acceptor complex and the reduction of the effective quantum yield, which results in impairment of photosynthesis. This provides a clue for future flavonoid metabolic engineering to regulate leaf senescence in wheat (Naik et al., 2021). Our findings imply that opportunity exists to manipulate leaf senescence and N remobilization under different N fertilization rates with the identified metabolites, which could be validated and used as biomarkers to select wheat cultivars with optimum leaf senescence and N remobilization.

## Conclusion

The present study was undertaken to profile metabolic changes in flag leaves sampled from spring-cultivated wheat under three regimes of N fertilization in our attempt to understand leaf senescence and N remobilization by UPLC-ESI-MS/MS. The no-N condition induced flavonoids (cyanidin 3-O-rutinoside, peonidin 3-O-glucoside, rhoifolin/apigenin 7-O-neohesperidoside, and astragalin/kaempferol 3-O-glucoside) and other secondary metabolites in an attempt to cope with the N-deficient condition. These compounds could be useful in metabolite-assisted breeding through their use as metabolic biomarkers to elucidate and regulate leaf senescence in wheat.

## Data availability statement

The datasets presented in this study can be found in online repositories. The names of the repository/repositories and accession number(s) can be found in the article/**Supplementary Material**.

## Author contributions

LL: funding acquisition, conceptualization, and supervision. CD: resources and project administration. ZE: investigation and writing—original draft. LW and JX: methodology. ZE, CD, MZ, and AX: data collection. ZE, SA, and BK: formal analysis. LL, BK, and EB: writing—review and editing. All authors read and approved the final manuscript.
