# Supplementary material for: Widely untargeted metabolomic profiling unearths metabolites and pathways involved in leaf senescence and N remobilization in spring-cultivated wheat under different N regimes
Source: Front Plant Sci. 2023 May 16;14:1166933. doi: 10.3389/fpls.2023.1166933 (PMC10227437; doi:10.3389/fpls.2023.1166933)
Supplement: Supplementary file 5 [file DataSheet_5.docx]

**Supplementary Figure S1.** Confirmation of 826 metabolites detected in this study to five public databases: Kyoto Encyclopedia of genes and genomes (KEGG) both compound name and pathway ([Kanehisa et al. 2016](#_ENREF_3)),  Human Metabolome Database (HMDB) ([Wishart et al. 2018](#_ENREF_6)), class assignment and ontology prediction using mass spectrometry (CANOPUS) ([Dührkop et al. 2021](#_ENREF_2)), PubChem ([Kim et al. 2016](#_ENREF_4)), Japan Chemical Substance Dictionary (NIKKAJI)([Kimura and Kushida 2015](#_ENREF_5)), and Chemical Entities of Biological Interest (ChEBI) ([Degtyarenko et al. 2007](#_ENREF_1)).

**References**

Degtyarenko, K., P. De Matos, M. Ennis, J. Hastings, M. Zbinden, A. McNaught, R. Alcántara, M. Darsow, M. Guedj & M. Ashburner, 2007. ChEBI: a database and ontology for chemical entities of biological interest. Nucleic acids research 36(suppl_1):D344-D350.

Dührkop, K., L.-F. Nothias, M. Fleischauer, R. Reher, M. Ludwig, M. A. Hoffmann, D. Petras, W. H. Gerwick, J. Rousu, P. C. Dorrestein & S. Böcker, 2021. Systematic classification of unknown metabolites using high-resolution fragmentation mass spectra. Nature Biotechnology 39(4):462-471 doi:10.1038/s41587-020-0740-8.

Kanehisa, M., Y. Sato, M. Kawashima, M. Furumichi & M. Tanabe, 2016. KEGG as a reference resource for gene and protein annotation. Nucleic Acids Research 44(D1):D457-D462 doi:10.1093/nar/gkv1070.

Kim, S., P. A. Thiessen, E. E. Bolton, J. Chen, G. Fu, A. Gindulyte, L. Han, J. He, S. He & B. A. Shoemaker, 2016. PubChem substance and compound databases. Nucleic Acids Research 44(D1):D1202-D1213.

Kimura, T. & T. Kushida, 2015. Openness of Nikkaji RDF data and integration of chemical information by Nikkaji acting as a hub. J Inf Process Manag 58(3):204-212.

Wishart, D. S., Y. D. Feunang, A. Marcu, A. C. Guo, K. Liang, R. Vázquez-Fresno, T. Sajed, D. Johnson, C. Li & N. Karu, 2018. HMDB 4.0: the human metabolome database for 2018. Nucleic Acids Research 46(D1):D608-D617.
