# Supplementary material for: Widely untargeted metabolomic profiling unearths metabolites and pathways involved in leaf senescence and N remobilization in spring-cultivated wheat under different N regimes
Source: Front Plant Sci. 2023 May 16;14:1166933. doi: 10.3389/fpls.2023.1166933 (PMC10227437; doi:10.3389/fpls.2023.1166933)
Supplement: Supplementary file 6 [file DataSheet_6.docx]

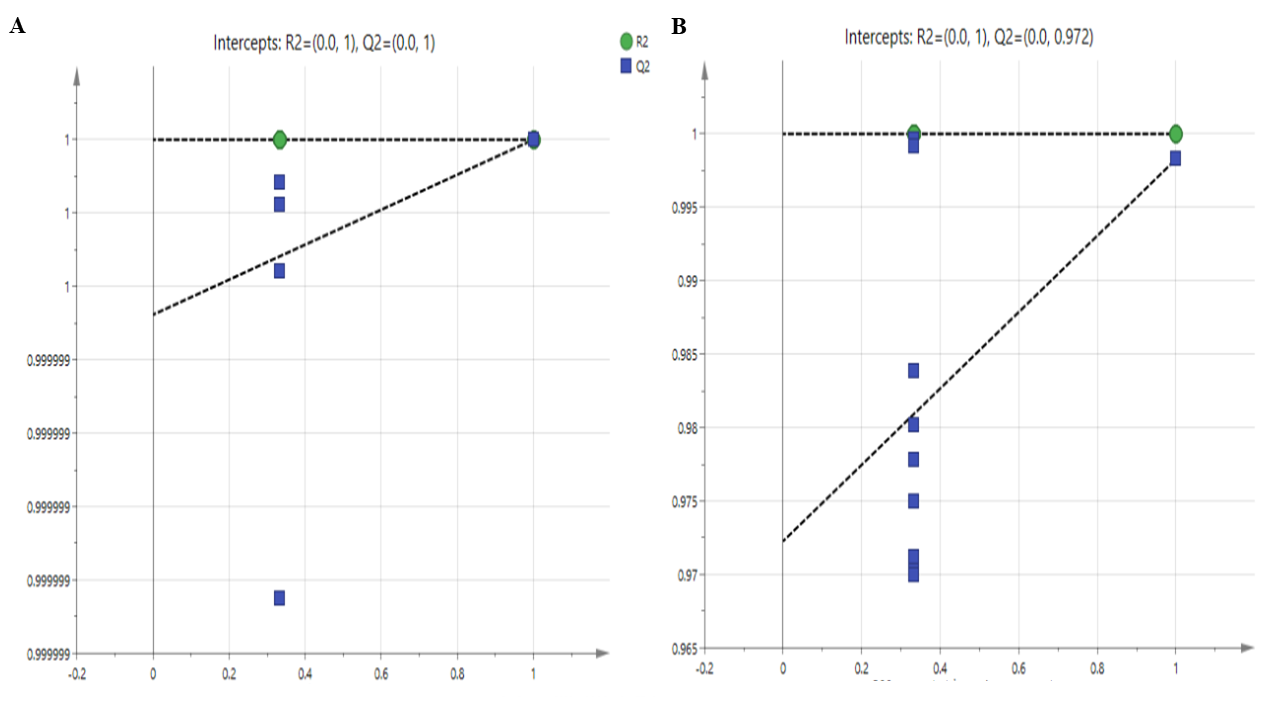


**Supplementary Figure S2.** Orthogonal projections to latent structures-discriminant analysis (OPLS-DA) of metabolites detected in the three samples from No nitrogen (G 1) relative to either Low nitrogen (G 2) or High nitrogen (G 3). The R2 obtained after cross-validation (model to categorical variable Y) and Q2 (predictability of the model) to judge the validity of the model. A. G 1_vs_G 2. B. G 1_vs_G 3.
